# An integrative *in silico *approach for discovering candidates for drug-targetable protein-protein interactions in interactome data

**DOI:** 10.1186/1471-2210-7-10

**Published:** 2007-08-20

**Authors:** Nobuyoshi Sugaya, Kazuyoshi Ikeda, Toshiyuki Tashiro, Shizu Takeda, Jun Otomo, Yoshiko Ishida, Akiko Shiratori, Atsushi Toyoda, Hideki Noguchi, Tadayuki Takeda, Satoru Kuhara, Yoshiyuki Sakaki, Takao Iwayanagi

**Affiliations:** 1 PharmaDesign, Inc., 2-19-8 Hatchobori, Chuo-ku, Tokyo, 104-0032, Japan; 2 Central Research Laboratory, Hitachi, Ltd., 1-280 Higashi-koigakubo, Kokubunji-shi, Tokyo, 185-8601, Japan; 3 Genomic Sciences Center, RIKEN, 1-7-22 Suehiro-cho, Tsurumi-ku, Yokohama, Kanagawa, 230-0045, Japan; 4 Graduate School of Genetic Resources Technology, Kyushu University, 6-10-1 Hakozaki, Higashi-ku, Fukuoka, 812-8581, Japan; 5 Research & Development Group, Hitachi, Ltd., 1-6-1 Marunouchi, Chiyoda-ku, Tokyo, 100-8220, Japan

## Abstract

**Background:**

Protein-protein interactions (PPIs) are challenging but attractive targets for small chemical drugs. Whole PPIs, called the 'interactome', have been emerged in several organisms, including human, based on the recent development of high-throughput screening (HTS) technologies. Individual PPIs have been targeted by small drug-like chemicals (SDCs), however, interactome data have not been fully utilized for exploring drug targets due to the lack of comprehensive methodology for utilizing these data. Here we propose an integrative *in silico *approach for discovering candidates for drug-targetable PPIs in interactome data.

**Results:**

Our novel *in silico *screening system comprises three independent assessment procedures: i) detection of protein domains responsible for PPIs, ii) finding SDC-binding pockets on protein surfaces, and iii) evaluating similarities in the assignment of Gene Ontology (GO) terms between specific partner proteins. We discovered six candidates for drug-targetable PPIs by applying our *in silico *approach to original human PPI data composed of 770 binary interactions produced by our HTS yeast two-hybrid (HTS-Y2H) assays. Among them, we further examined two candidates, RXRA/NRIP1 and CDK2/CDKN1A, with respect to their biological roles, PPI network around each candidate, and tertiary structures of the interacting domains.

**Conclusion:**

An integrative *in silico *approach for discovering candidates for drug-targetable PPIs was applied to original human PPIs data. The system excludes false positive interactions and selects reliable PPIs as drug targets. Its effectiveness was demonstrated by the discovery of the six promising candidate target PPIs. Inhibition or stabilization of the two interactions may have potential therapeutic effects against human diseases.

## Background

Most proteins exhibit their biological function via interactions with partner proteins, and thus, PPIs play fundamental and key roles in various cellular processes in organisms. PPIs have recently been recognized as challenging but attractive targets for small chemical drugs [1]. In particular, the inhibition of PPIs by SDCs has been intensively studied [1-5]. Investigations to date suggest that PPI inhibition by SDCs could lead treatments for some human diseases [1-5]. One of the well-investigated target PPIs is the interaction between tumor suppressor protein p53 and murine double-minute-2 protein (MDM2) [6-8]. It has been shown that a family of SDCs, the nutlins, inhibit this interaction [6,7], suggesting that the nutlins could be potential therapeutic drugs for cancer [8]. Several promising PPIs have been targeted by SDCs, such as AMAP1/cortactin for preventing breast cancer invasion and metastasis [9], B7.1/CD28 for modulating T-cell activation [10], BAK/BCL2 or BAK/BCL-X_L _for inducing apoptosis in tumor cells [11-14], β-catenin/Tcf4 for cancer treatment [15,16], IL2/IL2Rα for suppressing autoimmune diseases [17,18], LFA1/ICAM1 for modulating lymphocyte and immune system function [19-21], and NGF/p75^NTR ^for blocking neuropathic and inflammatory pain [22].

Although the PPIs targeted in the previous studies [6-22] were arbitrarily chosen according to the researchers' own interest in each individual PPI and by their interest in diseases related to the PPI, there have been few studies aimed at discovering or selecting target PPIs at the level of whole PPIs, called the 'interactome'. One reason for this has been the lack of strategies for comprehensively exploring and discovering target PPIs in the interactome. The enormous amounts of PPI data produced by HTS technologies in recent years [23-35] provide a promising opportunity for addressing this matter.

Here we propose a novel and integrative *in silico *approach for discovering candidates for drug-targetable PPIs by computationally screening large amounts of PPI data. To begin with, this approach is applied to the previously-investigated target PPIs, then the effectiveness and potential of the approach is demonstrated by applying the methodology to original human PPI data produced by our HTS-Y2H assays.

## Results

### Synopsis of our *in silico *system

Many previously-investigated target PPIs satisfy several criteria sufficient to be chosen as drug targets. One criterion is that interacting domains involved in a PPI have been already identified. Domain-domain interactions responsible for PPIs are more informative for researchers than PPIs to select potential drug targets [36]. This is because two domains that exclusively interact with each other can be specifically inhibited by a SDC without other PPIs being inhibited. In contrast, if a domain targeted by a SDC is shared with a large number of interacting proteins, and if this domain interacts with other domains, it is likely that the SDC will cause an off-target effect by inhibiting non-targeted PPIs that are essential to the organism.

A second criterion is the presence of SDC-binding pockets on the surface of the interacting protein. In many cases of the previously-investigated target PPIs, SDCs interact with a pocket in which the small number of amino acid residues exist that contribute the large fraction of protein-protein binding free energy, so-called 'hot spots' [1,37]. In order to inhibit a PPI by SDCs, one or both of the two interacting proteins should have a pocket on protein surface to which SDCs can bind. This criterion holds whether the SDCs exhibit their inhibiting effects via direct binding to the PPI interface, or via allosteric effects caused by SDC-induced conformational change to the tertiary structure of the SDC-interacting protein.

A third criterion is that the biological roles of the PPI are well understood. This is necessary in order to infer the phenotypic effects caused by inhibition of the PPI in the cell. In addition, if the two interacting proteins detected in an experimental study have the same cellular location and/or have similar biological functions, it is more probable that the interaction between these two proteins actually occurs in living cells.

Based on the idea of the *in silico *structure-based drug design, our novel and integrative *in silico *system discovers candidates for drug-targetable PPIs satisfying the above-mentioned criteria by integrating three independent assessment procedures:

• detection of protein domains responsible for PPIs,

• finding SDC-binding pockets on protein surfaces,

• evaluating similarities in the assignment of GO terms between specific partner proteins.

**Figure 1:**
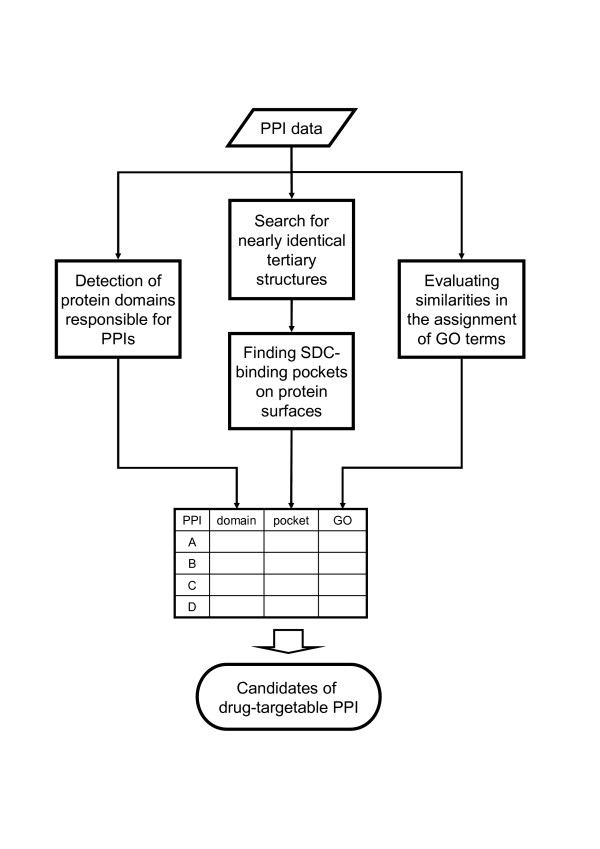
Schematic representation of our novel and integrative *in silico *system for discovering candidates for drug-targetable PPIs in binary PPI data. The system uses binary PPI data as an input and assesses each PPI based on three independent *in silico *investigations; detection of protein domains responsible for PPIs, finding SDC-binding pockets on protein surfaces, and evaluating similarities in the assignment of GO terms. By integrating the results of these three investigations, the system discovers candidates for drug-targetable PPIs.

The *in silico *system is schematically represented in Figure 1. The first assessment procedure utilizes protein domain information in the Pfam [38] database. In the second assessment procedure, we use two programs, CASTp [39] and MOE Alpha Site Finder [40], to find SDC-binding pockets. Similarity scores for GO-term assignment between specific partner proteins are calculated in the third assessment procedure. Statistical significance of the scores is also evaluated. For more details of these methods, see Methods section. In the following studies, we investigate a suitable threshold in each assessment procedure by applying our system to the previously-investigated target PPIs. Then, our system is applied to original human PPI data composed of 770 unique binary interactions produced by our HTS-Y2H assays.

### Application of our system to the previously-investigated target PPIs

We conducted the three *in silico *analyses on the 15 previously-investigated target PPIs in [1,4]; AMAP1/cortactin [9], B7.1/CD28 [10], BAK/BCL2(BCL-X_L_) [11-14], β-catenin/Tcf4 [15,16], CCR5/Env [41], CD4/MHC class II [42], CRM1/Rev [43], EPO/EPOR [44], IL1α (IL1β)/IL1R type I [45], IL2/IL2Rα [17,18], iNOS/iNOS [46], LFA1/ICAM1 [19-21], Myc/Max [47], NGF/p75^NTR ^[22], and p53/MDM2 [6-8]. Table 1 summarizes the results (see Additional file 1 for the full results of the analyses). As shown in Additional file 1, all proteins in the target PPIs have one or more Pfam-A and/or Pfam-B domains. By searching the public domain-domain interaction databases, iPfam [48], InterDom [49], and DIMA [50], we identified interacting partner domains in most of the target PPIs (Table 1). We found one or more pockets on at least one of the two interacting proteins in most target PPIs. Evaluation of similarity scores for GO-term assignment indicates that many target PPIs have statistically significant (*P *< 0.05) scores in two out of the three GO categories, cellular component, molecular function, and biological process. Taken together, we adopted the following thresholds in the three assessment procedures of our system.

**Table 1:** Summary of results from the three analyses for the previously-investigated target PPIs

PPI	interacting Pfam domain	presence of pockets	GO		
			
			*S*_*i*_^*C*^	*S*_*i*_^*F*^	*S*_*i*_^*P*^
AMAP1/cortactin	PF00018/PF00018	-/no	8	6	0
B7.1/CD28	not identified	yes/yes	1	6	1
BAK/BCL2(BCL-X_L_)	PF00452/PF02180, PF00452/PF00452	yes/yes	187**	19* (10)	396** (372**)
β-catenin/Tcf4	PF00514/PF08347	yes/-	98*	23*	171**
CCR5/Env	not identified	-/no	60	1	8
CD4/MHC class II	PF00047/PF00993, PF00047/PF07654	yes/-	178**	30**	16
CRM1/Rev	not identified	yes/-	98*	10	115**
EPO/EPOR	PF00758/PF09067, PF00758/PF00041	yes/yes	1	6	10
IL1α (IL1β)/IL1R type I	PF00340/PF00047	yes/yes	8	11*	35**
IL2/IL2Rα	PF00715/PF00084	no/yes	1	6	88*
iNOS/iNOS	PF02898/PF02898, PF00258/PF00258, PF00258/PF00175, PF00258/PF00667, PF00667/PF00667, PF00667/PF00258, PF00667/PF00175, PF00175/PF00667, PF00175/PF00258, PF00175/PF00175	yes/yes	90*	122**	104**
LFA1/ICAM1	PF00092/PF03921	yes/yes	123**	15*	8
Myc/Max	PF00010/PF00010, PF02344/PF00010	no/no	98*	23*	133**
NGF/p75^NTR^	PF00243/PF00020	yes/no	0	11*	93**
p53/MDM2	PF08563/PF02201	-/yes	362**	36**	233**

In the column of 'interacting Pfam domain', interacting partner domains identified by iPfam are shown. 'Domain a/domain b' in a row of a PPI shown by 'protein A/protein B' indicates that domain a in protein A and domain b in protein B interact with each other. In the column of 'presence of pockets', 'yes' means that one or more pockets were found by at least one of the two programs, 'no' means that no pocket was found, and '-' indicates that pockets were not searched because of lack of nearly identical tertiary structures. Statistical significance of similarity scores (*S*_*i*_^*C*^, *S*_*i*_^*F*^, and *S*_*i*_^*P*^) for GO-term assignment are indicated by '*' (* *P *< 0.05, ** *P *< 0.01). In the row of 'BAK/BCL2(BCL-X_L_)', similarity scores for BAK/BCL-X_L _are shown in parentheses.

• A domain pair in the PPIs has been already known or predicted as interacting partner in the public databases.

• One or both proteins have at least one pocket on the protein surface to which SDCs can bind.

• Similarity score for the GO-term assignment is statistically significant (*P *< 0.05) in two out of the three GO categories.

By adopting the thresholds, our system can select 8 PPIs (BAK/BCL2(BCL-X_L_), β-catenin/Tcf4, CD4/MHC class II, IL1α(IL1β)/IL1R type I, iNOS/iNOS, LFA1/ICAM1, NGF/p75^NTR^, and p53/MDM2) from the 15 previously-investigated target PPIs. In addition, the locations of the pockets found on the 8 PPIs are in good agreement with those of pockets targeted by SDCs in the previous studies (data not shown). Thus, we consider the thresholds to be suitable for assessing drug-targetability of each PPI, although some PPIs may be missed as false negatives.

### Application to original human PPI data

Most PPIs in original human PPI data are those between human transcription factors (baits) and other proteins (preys) (see Additional file 2). The number of unique baits and preys are 99 and 738, respectively (Table 2). The baits and preys used in our HTS-Y2H assays were sequence fragments. Protein domains included in the bait and prey fragments are likely involved in the interaction between the two fragments. All domains in the bait and prey fragments used in the present study were retrieved from the Pfam database (see Methods). We identified Pfam-A and/or Pfam-B domains in most of the bait (98% (97/99)) and prey (97% (714/738)) fragments (Table 2). Table 3 indicates that in most (95% (734/770)) bait-prey pairs, both fragments have Pfam-A and/or Pfam-B domains. This table also shows that only 3% (23/770) of bait-prey pairs satisfy the first criterion of our system, dramatically reducing candidate PPIs. Then, we further identified two domains as interacting partner domains, when a single domain was present in the bait fragment and a single domain in the prey fragment. Among the bait and prey fragments with domains, 32 (33%) bait and 350 (49%) prey fragments have a single domain. In 62 (8%) out of the 734 bait-prey pairs, we detected a single domain in both the bait and the prey fragments. As a result, we identified interacting partner domains in 83 (11%) bait-prey pairs. It is highly probable that these domain pairs are involved in the interaction between the bait and prey fragments. See Additional file 2 for the full list of the detected domains in the fragments.

**Table 2:** Summary of results from the three analyses for the bait and prey fragments

	bait	prey
# of unique fragments	99	738
# of fragments with Pfam domains	97	714
single domain	32	350
two or more domains	65	364
# of fragments with nearly identical tertiary structures	15	51
# of fragments with pockets	15	43
# of fragments with GO terms	97	672
cellular component	91	600
molecular function	93	635
biological process	89	591

**Table 3:** Summary of results from the three analyses for the bait-prey pairs

	bait-prey
# of unique pairs	770
# of pairs in which both fragments have domains	734
# of pairs in which a domain pair has been already known or predicted as interacting partner	23
# of pairs in which both fragments have a single domain	62
# of pairs satisfying one or both criteria for domain detection above	83
# of pairs in which one or both fragments have nearly identical tertiary structures	211
# of pairs in which one or both fragments have pockets	203
# of pairs with identical GO terms in any of the three categories	696
cellular component	603 (264)*
molecular function	647 (181)*
biological process	594 (256)*
# of pairs with statistically significant (*P *< 0.05) similarity scores for GO-term assignment in two out of the three GO categories	201

* Numbers of pairs with statistically significant (*P *< 0.05) similarity scores are shown in parentheses.

In order to computationally detect pockets on the surfaces of domains/proteins in the bait and prey fragments, it is essential that tertiary structures nearly identical to the bait and prey fragments are available. To detect protein tertiary structures nearly identical to the fragments, we searched for entries in the PDB [51] database showing high amino acid sequence identity and sequence coverage rate to the fragments (see Methods). The rigorous threshold of sequence identity ≥ 90% and coverage rate ≥ 90% in the results of sequence-similarity searches was adopted in the present study. This is because we detected pockets based on their volume and the number of hydrophobic amino acid residues in pockets, and these pocket properties are very sensitive to a slight conformational change of protein tertiary structure caused by amino acid replacement, deletion, or insertion. If sequence identity between a bait or prey fragment and a PDB entry fell within the range of 50%–90%, one could reconstruct a tertiary structure of the protein with homology modeling based on the template structure of the PDB entry. In these situations, however, pocket properties on the reconstructed tertiary structure would be not always nearly identical to those on the template structure. Therefore, we adopted the rigorous threshold of sequence identity ≥ 90% and coverage rate ≥ 90% for pocket detection. Results of the sequence-similarity search indicate that 15% (15/99) of bait and 7% (51/738) of prey fragments have nearly identical tertiary structures in the PDB database (Table 2). Most of the bait and prey fragments (100% (15/15) in bait, 84% (43/51) in prey) have one or more pockets on their protein surface. Table 3 shows that one or both fragments in 27% (211/770) of bait-prey pairs have nearly identical tertiary structures. In 96% (203/211) of the bait-prey pairs, we found SDC-binding pockets in one or both fragments. See Additional file 2 for the full results of the pocket analyses.

GO [52] is useful for assessing the biological significance of the bait-prey pairs and for selecting well-studied pairs. This is due to the hierarchical data structure of GO in which many biological terms are highly systematically organized to allow the computational handling of many terms related to biology. We counted the numbers of shared identical GO terms and calculated similarity scores between the bait and prey fragments (see Methods). Table 2 shows that most bait proteins (> 90%) and many prey ones (> 80%) have at least one GO term in any of the three GO categories. Table 3 indicates that many bait-prey pairs (> 75%) share one or more identical GO terms. We calculated similarity scores and evaluated statistical significance of the scores based on frequency distributions of scores calculated for PPI data composed of random protein pairs (see Additional file 3). The number of bait-prey pairs with a statistically significant (*P *< 0.05) score is shown in Table 3. Among these pairs, 201 bait-prey pairs have the statistically significant scores in two out of the there GO categories. See Additional file 2 for similarity scores calculated for all bait-prey pairs and results of the statistical evaluation of these scores.

**Figure 2:**
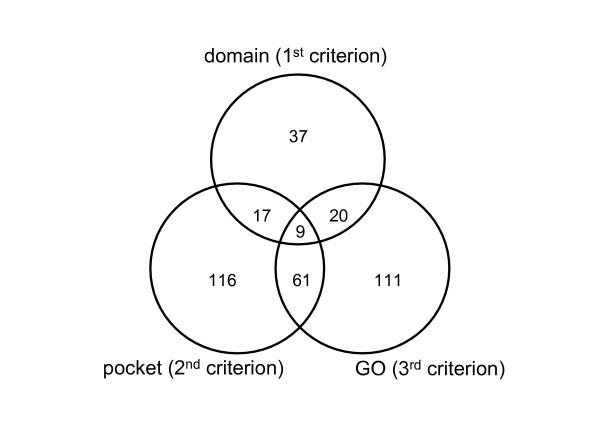
Selecting candidates for drug-targetable PPIs. Numbers of the bait-prey pairs satisfying one, two, or three criteria are shown in each region. Nine bait-prey pairs satisfy all the three criteria.

Among the 770 unique bait-prey pairs, we selected candidates for drug-targetable PPIs that satisfy all the three criteria. As shown in Table 3, 83 bait-prey pairs satisfied the first criterion. The number of bait-prey pairs satisfying the second or third criterion was 203 or 201, respectively. Figure 2 illustrates the distribution of the bait-prey pairs satisfying one, two, or three criteria described above. Twenty-six bait-prey pairs satisfy the first and second criteria, 70 pairs the second and third ones, and 29 pairs the first and third ones. Nine bait-prey pairs (6 protein pairs; RXRA/NRIP1, PPARA/RXRA, RXRB/PPARD, STAT1/STAT6, CDK2/CDKN1A, and STAT3/DST) were discovered as candidates for drug-targetable PPIs satisfying all the three criteria.

## Discussion

### Drug-targetability of selected PPIs

In this section, we discuss the drug-targetability of the two candidate PPIs, retinoid × receptor α (RXRA)/nuclear receptor-interacting protein 1 (NRIP1) and cell division protein kinase 2 (CDK2)/cyclin-dependent kinase inhibitor 1 (CDKN1A) (Table 4). The two candidates were selected, because both bait and prey fragments had a single domain, and interacting partner domains were explicitly determined, and because similarity scores for GO-term assignment were statistically significant in all the three GO categories. We further examined the two candidates with respect to their biological roles, PPI network around each candidate, and tertiary structures of the interacting domains.

**Table 4:** Two promising candidates for drug-targetable PPIs

	bait	prey	bait	prey
protein name	RXRA	NRIP1	CDK2	CDKN1A
full length of amino acid sequence	462	1158	298	191
N terminus of fragment	212	641	5	1
C terminus of fragment	462	1081	298	119
Pfam domain	PF00104 (Hormone_recep)	PB064381	PF00069 (Pkinase)	PF02234 (CDI)
Best match PDB entry				
PDB ID	1LBD	-	1V1K_A	-
presence of pockets	yes	-	yes	-
Similarity score for GO-term assignment				
*S*_*i*_^*C*^	98*		98*	
*S*_*i*_^*F*^	35**		31**	
*S*_*i*_^*P*^	164**		60*	

Statistical significance of similarity scores for GO-term assignment; * *P *< 0.05, ** *P *< 0.01.

#### RXRA/NRIP1

**Figure 3:**
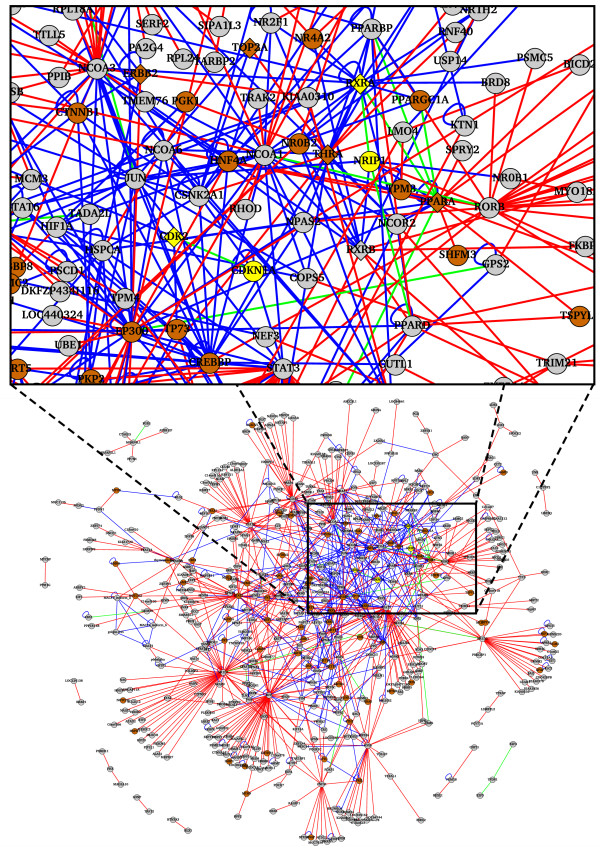
PPI network connecting proteins used in the HTS-Y2H assays in the present study. Part of the network around the RXRA/NRIP1 and CDK2/CDKN1A interactions is enlarged in the upper frame. Proteins are represented as diamonds (targets of drugs approved by FDA) and circles (non-targets of FDA-approved drugs). The information on target proteins of FDA-approved drugs was obtained from the DrugBank database [58]. RXRA, NRIP1, CDK2, and CDKN1A are colored yellow. Proteins related to OMIM [96] diseases are colored brown and the remaining proteins are grey. Interactions between proteins are indicated by lines. Novel PPIs detected in this study are shown in red, and those retrieved form a public database, HPRD [57], are in blue. PPIs are colored green if the interaction was detected in the present study and also retrieved from the HPRD. The network was drawn using the program Cytoscape (version 2.3.2) [97]. See Additional file 4 for the original and larger version of the PPI network.

Biological functions of RXRA and NRIP1 have been studied in detail [53-56]. The statistically significant similarity scores for the GO-term assignment indicate that RXRA and NRIP1 have related biological functions (Table 4). In fact, the two proteins share a number of gene-transcription-related GO terms; 'nucleus' in the cellular component category, 'transcription coactivator activity' and 'DNA binding' in the molecular function category, and 'regulation of transcription, DNA-dependent' and 'positive regulation of transcription from RNA polymerase II promoter' in the biological process category. RXRA is a member of the nuclear hormone receptor family. When a ligand binds to its hormone receptor domain, RXRA forms a homo- or hetero-dimer with other nuclear hormone receptors in order to function as a transcription factor [56]. NRIP1 interacts with homo- or hetero-dimers of various nuclear hormone receptors and modulates their function by repressing transcriptional activity of the dimers [53-55]. Figure 3 shows the interaction network based on PPI data originally produced by our HTS-Y2H assays and retrieved from a public PPI database, HPRD [57] (see Additional file 4 for the original and larger version of Figure 3). The network shows that RXRA interacts with proteins related to a tumor (THRA related to pituitary adenome) and those related to certain diseases caused by abnormalities in lipid metabolism (*e.g*., NR0B2 related to obesity, PPARA to hyperapobetalipoproteinemia, and PPARGC1A to lipodystrophy). Among the proteins interacting with RXRA and NRIP1, several proteins (*e.g*., PPARA, THRA, RARG, and RXRA itself) are targeted by the drugs approved by the Food and Drug Administration (FDA) [58]. Indeed, members of the nuclear hormone receptor family, including RXRA, have been intensively studied as targets for therapeutic drugs for human diseases such as type II diabetes, obesity, and cancer [56]. Considering the biological functions of RXRA and NRIP1, we speculate that SDCs inhibiting the RXRA/NRIP1 interaction may have an effect similar to that of a RXRA agonist. If inhibition of the RXRA/NRIP1 interaction by the SDCs results in NRIP1 separating from a protein complex composed of RXRA, another nuclear receptor, and NRIP1, the transcription factor functionality of the resulting dimer would be restored.

**Figure 4:**
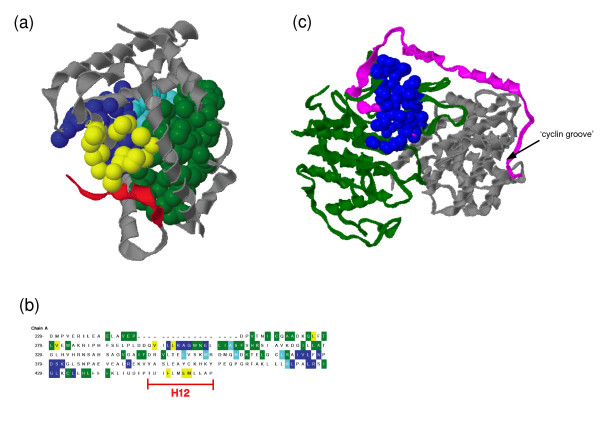
Locations of the detected pockets superimposed on the tertiary structures of proteins or the amino acid sequence. (a) The main chain of Hormone_recep domain of RXRA [PDB:1MVC_A] is shown by a ribbon model and is colored grey. Atoms of the four detected pockets are shown as space-filling models, and each pocket is colored green (1,092Å^3^, 64%), blue (463Å^3^, 48%), light blue (169Å^3^, 82%), or yellow (152Å^3^, 78%), (the volume and the hydrophobic residue ratio of each pocket are shown in parentheses). The H12 region is shown in red. (b) Amino acid sequence of the Hormone_recep domain of RXRA [PDB:1MVC_A]. Amino acid residues comprising each pocket are color-coded as in (a). (c) Location of a pocket laid across CDK2 and CDKN1B on CDK2/CDKN1B/cyclin A complex [PDB:1JSU]. CDK2, CDKN1B, and cyclin A are shown in green, magenta, and grey, respectively. Atoms are colored blue composing of the pocket with the size of 714Å^3 ^and the hydrophobic residue ratio of 50%. The location of the 'cyclin groove' [62] already studied as drug target is also shown. The figures were drawn using the CASTp [39].

We identified interaction between the Hormone_recep domain (ligand-binding domain) [Pfam:PF00104] in RXRA and a fragment of the PB064381 domain containing LXXLL motifs in NRIP1 (Table 4). The RXRA/NRIP1 interaction is believed to occur between α-helix 12 (H12) located in the C-terminal region of the Hormone_recep domain in RXRA and the LXXLL motifs in NRIP1 [54,55]. Since RXRA interact with NRIP1 in a ligand-dependent manner [53-55], one would expect to detect pockets on the surface of RXRA in the ligand-bound state. 1LBD in Table 4, however, is not suitable for the present study because it is the tertiary structure of RXRA homo-diners in the non-ligand-bound state. Then, we further detected pockets on 1MVC_A (RXRA in the ligand-bound state) with the second-highest score to the bait fragment from RXRA in the sequence similarity search. Figure 4(a) and 4(b) show the locations of the found pockets and of the H12 from the Hormone_recep domain superimposed on the tertiary structure of 1MVC_A. We found four pockets using CASTp and three using MOE Alpha Site Finder on the surface of the Hormone_recep domain in RXRA. The pockets range in size from 152Å^3 ^to 1,092Å^3^. The ratio of the number of hydrophobic amino acid residues to that of total residues was calculated for each pocket, ranging from 48% to 82%. The pocket with the size of 152Å^3 ^and 78% hydrophobic residues (shown in yellow in Figure 4(a)) seems most adequate for SDCs designed to inhibit RXRA/NRIP1 interaction, because several amino acid residues in the pocket are shared with the H12 (Figure 4(b)). Based on this structural information, it may be possible to discover inhibitors of the RXRA/NRIP1 interaction by designing SDCs to specifically bind to the pocket. Peptidomimetics of the LXXLL motif [5] in NRIP1 could be used as templates for designing RXRA/NRIP1-inhibiting drugs. In addition, the PB064381 domain is unique to NRIP1 [59], suggesting that inhibition of the Hormone_recep/PB064381 interaction may not affect other domain-domain interactions in living cells.

#### CDK2/CDKN1A

CDK2 and CDKN1A share several GO terms; 'nucleus' in the cellular component category, 'protein kinase activity' and 'protein binding' in the molecular function category, and 'cell cycle' in the biological process category. This indicates that the both proteins have biological functions in signaling pathways related to cell cycle regulation in the nucleus. CDK2 forms a protein complex with a member of cyclin family proteins, and functions in cell cycle progression at the transition between the G1 and S phases [60]. CDKN1A arrests cell cycle progression by acting as an inhibitor of CDK2/cyclin protein complex [61]. The PPI network illustrated in Figure 3 shows that CDK2 interacts with the TP73 protein related to neuroblastoma. Like the RXRA, the CDK family proteins have attracted the researchers' interest as targets for anticancer drugs [62-64]. A large number of SDCs have been developed that interact with ATP-binding pocket and inhibit CDKs' kinase activity [63,64]. Likewise, CDK/cyclin protein complexes have well studied as therapeutic target [62]. CDKN1A represses CDK2/cyclin activity by simultaneously binding to the 'cyclin groove' on cyclin and ATP-binding pocket on CDK2 [61,62], which suggests that CDKN1A has an effect similar to that of an antagonist of CDK2's kinase activity. Indeed, Kontopidis and his colleagues have obtained some peptides that mimic cyclin-groove-binding motif in CDKN1A and inhibit interaction between CDK/cyclin complex and transcription factors [62]. In addition to these peptidomimetics of CDKN1A, SDCs, called 'dimerizers' [65], that induce or stabilize CDK2/cyclin A/CDKN1A protein complex could potentially lead to treatments for cancer.

We identified domain-domain interaction between the Pkinase domain [Pfam:PF00069] in CDK2 and the CDI domain [Pfam:PF02234] in CDKN1A (Table 4). This is in good agreement with the results in the previous studies [66] identifying interaction interface of CDK2/CDKN1A. One strategy for inducing or stabilizing a PPI is to design a SDC that can simultaneously bind to a pocket laid across two interacting proteins on a protein complex. In the case of CDK2/CDKN1A, we found pockets on the Pkinase domain [PDB:1V1K_A] in CDK2 but did not detect any pocket on the CDI domain in CDKN1A because it has no nearly identical tertiary structure (Table 4). Instead of 1V1K_A, we further investigated a tertiary structure of protein complex [PDB:1JSU] composed of CDK2, cyclin A, and CDKN1B that is a homolog of CDKN1A (sequence identity < 45%). Figure 4(c) shows that there is a pocket (shown in blue in Figure 4(c)) composed of atoms from CDK2 and from CDKN1B. Most of the atoms overlap with those composing ATP-binding pocket on CDK2. The size is 714Å^3^, and the ratio of hydrophobic residues in the pocket is 50%. It is highly probable that CDK2/CDKN1A complex has a tertiary structure not nearly identical but similar to CDK2/CDKN1B complex, and that CDKN1A binds to CDK2 in a similar mode to CDKN1B [67]. Therefore, we speculate that SDCs, that bind to the pocket and interact with atoms both from CDK2 and from CDKN1A, may stabilize the protein complex and become a candidate for anticancer drugs. Unlike the Hormone_recep/PB064381 interaction in RXRA/NRIP1, many human proteins share the Pkinase domain with CDK2 [68] and the CDI domain with CDKN1A [69]. Thus, less influence on other PPIs may be strongly required for SDCs that can specifically induce or stabilize Pkinase/CDI interaction in CDK2/CDKN1A.

### Advantages of targeting PPIs

Targeting PPIs has distinct advantages over targeting single proteins; a larger number of undiscovered potential drug targets. Using traditional approaches for drug target discovery from the human proteome, drug targets were single proteins and limited to a small number (~480) of proteins such as membrane receptors and enzymes [70]. Furthermore, most pockets targeted by small chemical drugs in these approaches were those to which endogenous small molecule ligands or substrates bind. By focusing on PPIs, the number of latent and novel drug targets can be expected to dramatically increase. This is because the size of the human interactome must be considerably larger than that of the human proteome and because many pockets involved in PPIs but not targeted in the traditional approaches become accessible. Since the total number of proteins encoded on the human genome is about 25,000 – 40,000, the size of the human interactome has been estimated to be 40,000 – 200,000 PPIs, based on extrapolation from the yeast interactome (10,000 – 30,000 PPIs (3 – 10 interactions/protein)) [71]. However, the number of human PPIs, registered in the public interaction database, is limited to ~38,000 [57]. Therefore, it is highly probable that most PPIs, including those which could be potential drug targets in the human interactome, remain undiscovered. For example, some PPIs, including BAK/BCL2, BAK/BCL-X_L_, p53/MDM2, and homo- or hetero-dimers of nuclear receptors, are mediated by hydrophobic grooves formed by three α-helices [1,56]. These PPIs utilizing α-helix grooves are thought to be amenable to small-molecule drug discovery [1], and thus may be promising targets of PPI-inhibiting SDCs [1,5].

Our *in silico *system can select more reliable interactions as drug targets by excluding spurious interactions via the three independent assessment procedures. PPI data used in the present study were obtained from our HTS-Y2H assays. In general, the false positive rate of HTS-Y2H methods has been believed to be higher than that of other physical, genetic, biochemical, or immunological methods for experimental detection of PPIs, mainly due to 'sticky' proteins that non-specifically interact with various proteins [72]. While a recent study on PPI prediction by the Support-Vector-Machine-based method has implied that PPI data produced by our HTS-Y2H assays are more reliable than data in the previous HTS-Y2H studies (Table 4 in [73]), we do not neglect the possibility that our PPI data also contain false positive interactions. Indeed, our HTS-Y2H assays identified PPIs between baits derived from nucleus-located proteins and preys from extracellular proteins such as collagen α-1(XV) chain (COL15A1), extracellular matrix protein 1 (ECM1), and laminin proteins (LAMA3, LAMB3, and LAMC2) (see Additional file 2). These PPIs are highly probable to be false positives. Our *in silico *system, however, can exclude these spurious interactions, because, in these cases, similarity scores for GO-term assignment are not statistically significant in the cellular component category. Therefore, our approach should be widely applicable to PPI data even if a number of false positive interactions are included.

### Issues in out approach

Our approach has some advantages described above, but some issues should be noted for further refinement of the approach. For more careful assessment of domain detection, we did not identify interacting partner domains when bait and/or prey fragments have multiple domains, so long as a domain pair was not registered in the public domain-domain interaction databases. However, a large number of human proteins are multi-domain ones, and this is also the case in the bait (> 60%) and prey (> 45%) fragments used in the present study. Several computational methods have been developed in recent years for predicting interacting partner domains from large amounts of experimental PPI data [74-80]. Application of the methods to the PPI data used in this study will be needed for more exhaustive identification of interacting domains. For the purpose of pocket detection, we adopted simple criteria mainly based on pocket volume and the number of amino acid residues composing the pocket. Many studies in past few decades have revealed various properties of pockets involved in endogenous ligand binding or PPI [[37,81-83] and references therein]. These properties, such as volume, shape, hydrophobic clusters, shallowness, roughness, and accessible surface area, can be taken into consideration as parameters for assessment of drug-targetability of each pocket. We are now developing a computer program that evaluates drug-targetability of pockets based on these parameters. The program will enable us to judge whether a pocket is suitable for drug target. To investigate whether biological function of each PPI has been well understood or not, we assessed each PPI by using GO terms. GO has been frequently used in PPI network studies for researchers' purpose of annotating biological function of PPIs [28-32,34], but it has also a weak point that well-studied proteins have many GO terms and poorly-understood ones have little. While PPIs between well-studied proteins have been annotated too much, those between poorly-understood ones too little. Thus, when our approach assesses PPIs by using GO terms, it may miss poorly-understood but therapeutically important target PPIs as false negatives. But, one of the aims of our system is to select PPIs on which biological information are more abundant. *In vivo *and *in vitro *validation process of PPIs as drug target, it is more desirable that a researcher can obtain as much information as possible on biology of the PPIs. Since PPIs annotated too little are considered as difficult target in this respect, our system does not select the PPIs in this study. More accumulation of GO annotation will help us select therapeutically important target PPIs that are annotated too little by GO terms at present.

### Future directions

Our *in silico *system can be further expanded for more precise assessment of candidates for drug-targetable PPIs if other computational methods are incorporated. These methods include the prediction of interaction interfaces on protein tertiary structures, the prediction of disordered regions, and the evaluation of similarities in the expression patterns of messenger RNAs encoding the two interacting proteins in every tissue/organ. In the case of RXRA/NRIP1 and CDK2/CDKN1A, it is fortunate that the interaction interfaces have been well studied by biochemical and immunological approaches [54,55,66], although the tertiary structures of the protein complexes remain unsolved. However, if the interaction interface of a candidate target PPI has not been well studied and the tertiary structure of the protein complex is unknown, computational methods to predict the PPI interface [84-88] are required in order to determine whether a detected SDC-binding pocket is located at the interface. Cheng and colleagues [89] recently proposed that interaction interface regions in proteins tend to have disordered tertiary structures and that information regarding these disordered regions is useful for drug target discovery. As for gene expression patterns, two proteins could presumably interact in living cells, if the expression patterns of their corresponding genes were similar to each other.

We focused on discovering drug targets for SDCs based on the idea of the structure-based *in silico *drug design, although there are various other types of drugs, including peptides, antisense RNAs or DNAs, aptamers, and antibodies. Candidate target PPIs for each type of drugs, as well as small chemical drugs, will be selected by adopting distinct criteria based on the three (or more) independent *in silico *investigations in our system. For example, to select candidate target PPIs for antibodies, one can adopt criteria so that i) at least one tertiary structure of the interacting domains is known, ii) the interacting domain has an interaction interface predicted to be recognized by antibodies, and iii) the interacting proteins share identical GO terms such as 'extracellular' in the cellular component category and have expression patterns similar to each other.

## Conclusion

In this paper, we propose a novel and integrative *in silico *approach for discovering candidates for drug-targetable PPIs in interactome data. The system excludes false positive interactions and selects more reliable PPIs as drug targets. The application of our system to original human PPI data demonstrated its effectiveness by discovering the six promising candidates for drug-targetable PPIs. Advances in HTS technologies for detecting PPIs and the accumulation of high fidelity PPI data in the near future will enable our system to facilitate the more comprehensive exploration of drug-targetable PPIs.

## Methods

### PPI data

The PPI data analysed in the present study consists of 770 binary interactions between human proteins. The data were produced by our HTS-Y2H assays supported by the Genome Network Project from the Ministry of Education, Culture, Sports, Science and Technology of Japan. See Additional file 2 and the website of the Genome Network Platform [90] for all PPI data used in this study. Most of bait proteins used in the HTS-Y2H assays are transcription factors, including members of the nuclear hormone receptor family (NR1D1, NR1D2, PPARA, PPARD, RORB, RXRA, THRA, *etc*), those of the Signal Transducer and Activator of Transcription (STAT) family (STAT1, STAT3, and STAT4), homeodomain proteins (FOXP2, LHX1, LHX2, PKNOX1, *etc*), and zinc-finger proteins (RFP, ZNF31, ZNF581, TRIM21, *etc*). Preys used in the assays were prepared from cDNA libraries derived from various cell lines (brain, breast cancer/prostate cancer, liver, and macrophage). Our HTS-Y2H method uses sequence fragments as baits, and preys isolated with the baits are also sequence fragments. This enables us to identify protein domains responsible for PPIs because it is highly probable that protein domains included in the bait or prey fragments are involved in the interactions between the two fragments. Full details of our HTS-Y2H method, including experimental materials and conditions, will be reported elsewhere in near future.

### Detection of protein domains responsible for PPIs

All domains in the bait and prey fragments were retrieved from the Pfam (version 20.0) database [38] using the UniProt (release 50.3) or TrEMBL (release 33.3) database [91] accession numbers associated to the fragments. When no domain was detected in a bait or prey fragment, the bait or prey fragment was further searched for Pfam domains to profile Hidden Markov Models of the Pfam-A and Pfam-B domains using the program HMMPFAM [92]. The HMMPFAM search was performed with the default program parameters except for '-E 0.1 – domE 0.1' (*E*-value < 0.1 for each detected domain). If the sequence length of a detected domain included in a fragment was < 10 residues, the domain was excluded in the following studies. To check whether a domain pair has been known or predicted as interacting partner in previous studies, all combinations of domains between bait and prey fragments were searched for the public domain-domain interaction databases, iPfam [48], InterDom version 1.1 [49], and DIMA [50].

### Finding SDC-binding pockets on protein surfaces

Using amino acid sequences of the bait and prey fragments as queries, we searched the PDB database [51] (the version at the date of 2006/5/18) for tertiary structures similar to each fragment using the program BLASTP (version 2.2.13) [93]. This similarity search was performed with the default program parameters except for '-F F' (no mask for low complexity regions) and '-e 0.001' (*E*-value < 0.001). We considered the fragment to have a tertiary structure nearly identical to the chain, when a bait or prey fragment had sequence identity of ≥ 90% and query coverage rate (length of query sequence showing the identity/full length of the query sequence) of ≥ 90% to a chain in a PDB entry, and if the sequence length showing the identity was ≥ 50 residues. If no nearly-identical tertiary structure was detected for a fragment, the fragment was further searched in the PDB database using the program PSI-BLAST (version 2.2.13) [93]. The default program parameters were used for the PSI-BLAST search except for '-j 10' (10 times the iteration search).

The search for pockets on protein surfaces was performed for the bait and prey fragments showing high sequence identity (≥ 90%) to a chain in a PDB entry. We used two programs, CASTp [39] and MOE Alpha Site Finder [40], which implement different pocket-search algorithms. Coordinate data for the chains in the PDB showing high sequence identity to the bait and prey fragments were used as input to the programs. We counted the number of pockets satisfying the following empirically-determined criteria in order to detect potential SDC-binding pockets: in the case of CASTp, i) the volume (*v*) of a detected pocket was within the range of 150Å^3 ^<*v *≤ 2000Å^3^; ii) in that of MOE Alpha Site Finder, a) the number of atoms comprising the side chains of the amino acids inside the pocket was ≥ 37 or b) the number of hydrophobic atoms inside the pocket was ≥ 22.

### Evaluating similarities in the assignment of GO terms between specific partner proteins

Based on GO terms assigned to two proteins from which the bait and prey fragments were derived, we evaluated similarities between fragments by counting the number of shared identical GO terms. GO terms assigned to the proteins were retrieved from the QuickGO database [94] using the UniProt/TrEMBL accession numbers. GO organizes a wide variety of biological terms as hierarchy. If a specific term is assigned to a gene product, then all 'parent' terms in all paths ascending from that specific term to the top level terms ('cellular component', 'biological process', and 'molecular function') of the hierarchy are also assigned to that gene product [96]. Thus, we collected all parent terms of specific ones assigned to each protein. A similarity score (*S*_*i*_) between a protein pair *i *is calculated as

Si=∑jLj⋅nij,
 MathType@MTEF@5@5@+=feaafiart1ev1aaatCvAUfKttLearuWrP9MDH5MBPbIqV92AaeXatLxBI9gBaebbnrfifHhDYfgasaacH8akY=wiFfYdH8Gipec8Eeeu0xXdbba9frFj0=OqFfea0dXdd9vqai=hGuQ8kuc9pgc9s8qqaq=dirpe0xb9q8qiLsFr0=vr0=vr0dc8meaabaqaciaacaGaaeqabaqabeGadaaakeaacqWGtbWudaWgaaWcbaGaemyAaKgabeaakiabg2da9maaqafabaGaemitaW0aaSbaaSqaaiabdQgaQbqabaGccqGHflY1cqWGUbGBdaWgaaWcbaGaemyAaKMaemOAaOgabeaaaeaacqWGQbGAaeqaniabggHiLdGccqGGSaalaaa@3E18@

where *L*_*j *_is the *j*th level of GO hierarchy (in the present study, *L*_*j *_= 1, 2, 3, ..., 13, from the top level term (*L*_*j *_= 1) to a specific term (*L*_*j *_> 1)) and *n*_*ij *_is the number of shared identical GO terms in the *j*th level between a protein pair *i*. We calculated the scores for the three GO categories; cellular component (*S*_*i*_^*C*^), molecular function (*S*_*i*_^*F*^), and biological process (*S*_*i*_^*P*^).

Statistical significance of the similarity scores was evaluated on the basis of frequency distributions of scores calculated for PPI data composed of 10,000 random pairs of human proteins (see Additional file 3). The random pairs were constructed from proteins in the UniProt and TrEMBL database with GO terms. The frequency distributions of random scores were calculated for all three GO categories, and probabilities of the real scores were estimated based on the distributions.

## Abbreviations

PPI, protein-protein interaction; HTS, high-throughput screening; SDC, small drug-like chemical; GO, Gene Ontology; HTS-Y2H, high-throughput screening yeast two-hybrid.

## Authors' contributions

NS conceived of the study, carried out the studies on domain detection and gene ontology, and drafted the manuscript. KI and TTashiro carried out the protein structure and pocket studies. ST, JO, YI, AS, AT, HN, TTakeda, and TI designed and carried out the HTS-Y2H assays. SK and YS conceived and supervised this study. All authors read and approved the final manuscript.

## Supplementary Material

Additional file 1 Full results of our analyses of the previously-investigated target PPIs. This file lists the previously-investigated target PPIs and summarizes the full results of domain detection, finding SDC-binding pockets, and evaluating similarities in GO-term assignment. Click here for file

Additional file 2 Full results of our analyses of original human PPI data. This XLS-format file lists original human PPIs analysed in the present study and summarizes the full results of domain detection, search for nearly identical tertiary structures and finding SDC-binding pockets, and evaluating similarities in GO-term assignment. Click here for file

Additional file 3 Frequency distributions of similarity scores for GO-term assignment calculated for random protein pairs. This file contains a figure illustrating frequency distributions of similarity scores for GO-term assignment calculated for PPI data composed of 10,000 random pairs of human proteins. Click here for file

Additional file 4 PPI network of original human PPI data. This file is an original version of the PPI network in Figure 3. For description of colors and shapes of nodes and colors of edges, see the legend to Figure 3. Click here for file
