# Supplementary figures and images for: An integrative in silico approach for discovering candidates for drug-targetable protein-protein interactions in interactome data
Source: BMC Pharmacol. 2007 Aug 20;7:10. doi: 10.1186/1471-2210-7-10 (PMC2045083; doi:10.1186/1471-2210-7-10)

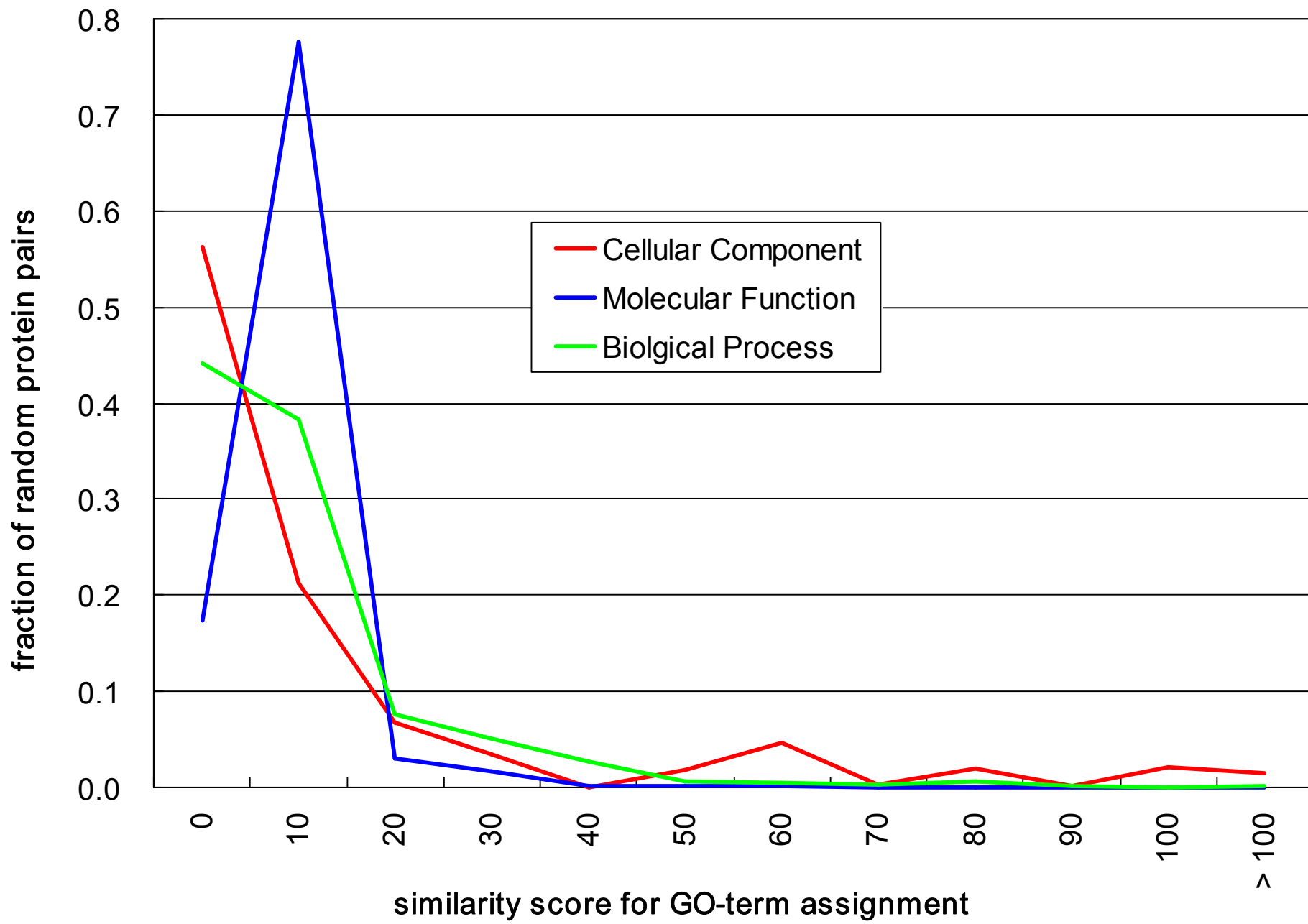

Supplement: Additional file 3 — Frequency distributions of similarity scores for GO-term assignment calculated for random protein pairs. This file contains a figure illustrating frequency distributions of similarity scores for GO-term assignment calculated for PPI data composed of 10,000 random pairs of human proteins. [file 1471-2210-7-10-S3.pdf]

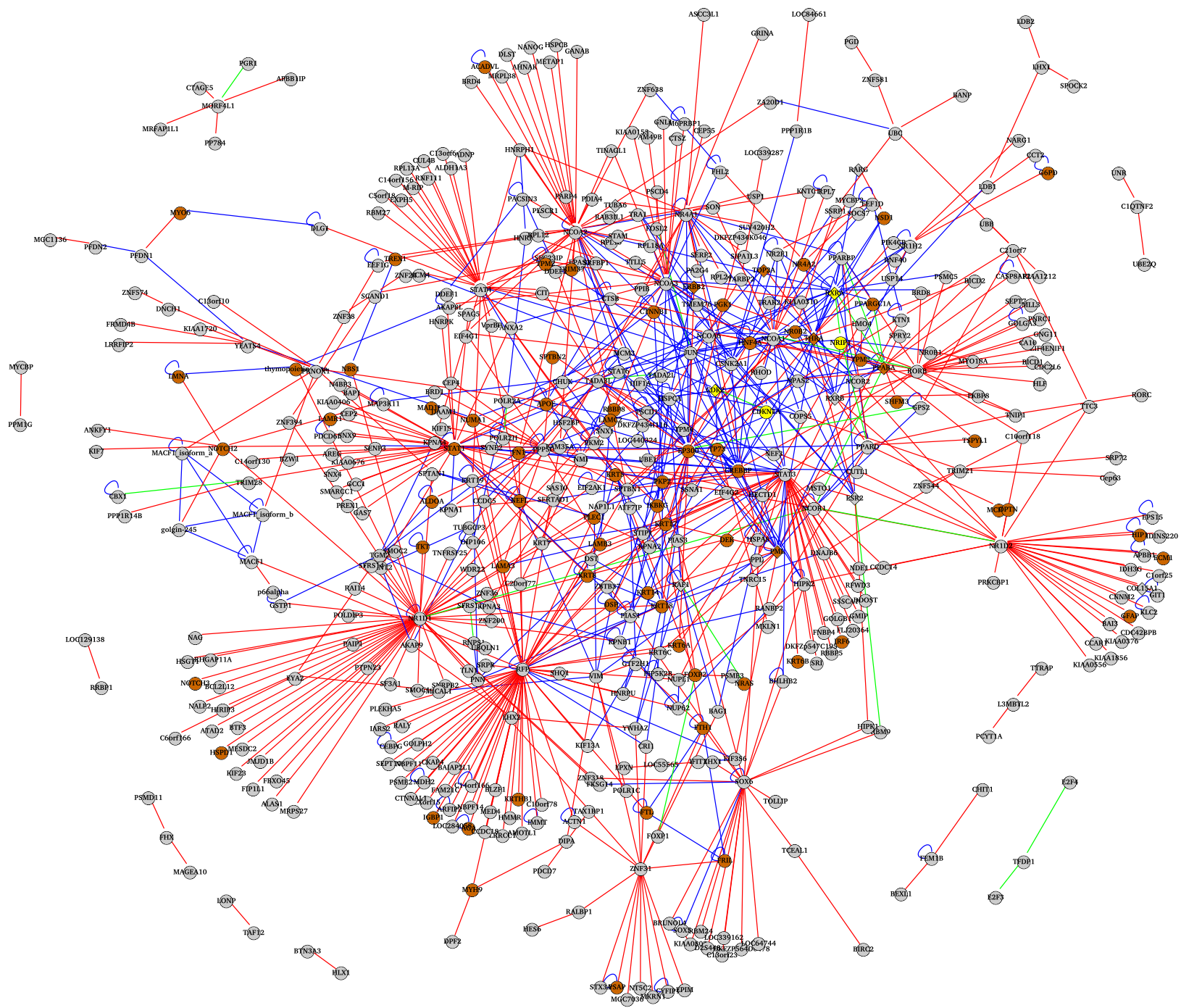

Supplement: Additional file 4 — PPI network of original human PPI data. This file is an original version of the PPI network in Figure 3. For description of colors and shapes of nodes and colors of edges, see the legend to Figure 3. [file 1471-2210-7-10-S4.pdf]
